# Supplementary material for: Susceptibility to Pentylenetetrazole-Induced Seizures in Mice with Distinct Activity of the Endogenous Opioid System
Source: Int J Mol Sci. 2024 Jun 26;25(13):6978. doi: 10.3390/ijms25136978 (PMC11241619; doi:10.3390/ijms25136978)
Supplement: Supplementary file 1 [file ijms-25-06978-s001.zip › ijms-3010917-supplementary.pdf]

International Journal of
Molecular Sciences

Supplementary Materials

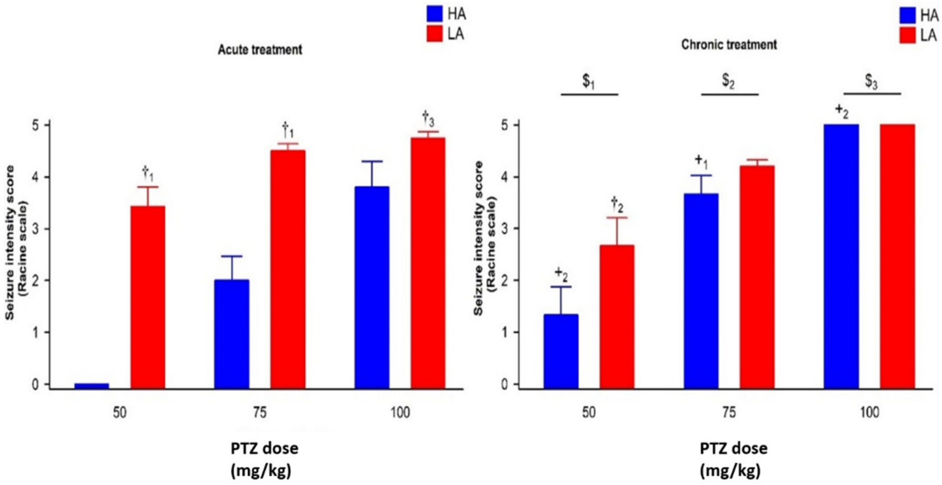

Figure S1. Seizure intensity according to Racine's scale assessed in HA and LA mice in kindling model (n = 8–10). Animals were kindled with 20 mg/kg of PTZ for 15 days and injected with 50, 75 or 100 mg/kg of PTZ on the last day of experiment (day 16). HA and LA mice were grouped as per distinct PTZ dosage and line type (n = 8–10 per one subgroup). '†' symbol denotes significance of comparisons among HA vs. LA mice. Analyses considered different doses of PTZ and comparisons between mouse lines were performed within groups receiving the same dose of the compound. '†' denotes significance of post-hoc comparisons between subgroups of HA and LA mice for each PTZ dose (to analyse epileptogenic effect of PTZ). '+' denotes significance of post-hoc comparisons within HA or LA lines and between subgroups subjected to acute or chronic administration of PTZ (repeated PTZ dosing to modify seizures intensity). '$' denotes significance of comparisons among HA and LA individuals considering the influence of multiple PTZ injections (effect of interaction between mouse line type and PTZ treatment regimen). Subscripts 1, 2 and 3 denotes significance of comparisons fixed at 0.001; 0.01 and 0.05, respectively.

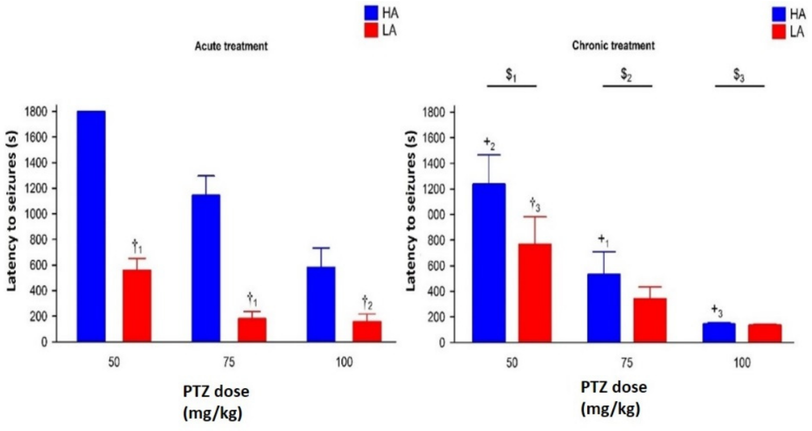

Figure S2. Seizure threshold measured in LA and HA mice following chronic ip. administration of PTZ at doses of 50, 75 and 100 mg/kg (n = 8–10). HA and LA mice were divided into subgroups as per distinct PTZ dosage and line type (n = 8–10 per one subgroup). '†' symbol denotes significance of comparisons among HA vs. LA mice. Analysis considered different doses of PTZ and comparisons between mouse lines were performed within groups receiving the same dose of the compound. '†' denotes significance of post-hoc comparisons between subgroups of HA and LA mice for each PTZ dose (epileptogenic effect of PTZ). '+' denotes significance of post-hoc comparisons within HA or LA lines and between subgroups subjected to acute or chronic administration of PTZ (modification of seizures intensity by repeated injections with PTZ). '$' denotes significance of comparisons among HA and LA individuals considering the influence of multiple PTZ injections

(effect of interaction between mouse line type and PTZ treatment regimen). Subscripts 1, 2 and 3 denotes significance of comparisons fixed at 0.001; 0.01 and 0.05, respectively.

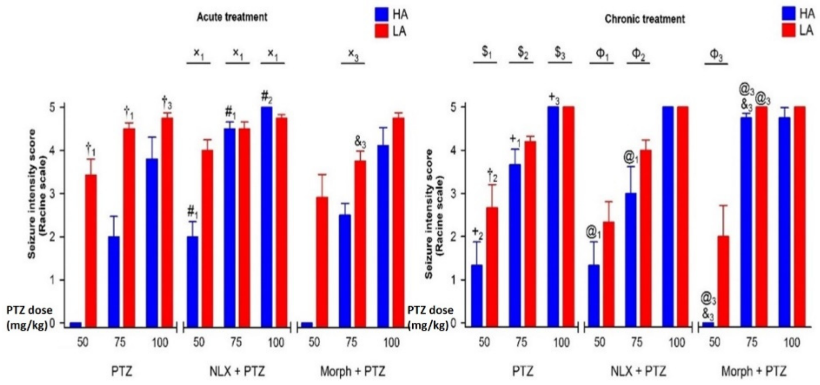

Figure S3. The effect of naloxone (NLX) and morphine (Morph) on seizure intensity produced by repeated PTZ administration (50, 75 and 100 mg/kg, ip.) in HA and LA mice (n = 8–10). Seizure intensity was assessed according to Racine's scale. NLX or morphine were given ip. 30 min. before PTZ injection at doses of 27.5 μmol/kg. Seizures were kindled every day by ip. injection of PTZ (20 mg/kg). Results were analyzed with two- or three-way ANOVA, followed by the Bonferroni's post-hoc test. The level of statistical significance was set to 0.05. Group comparisons were presented as follows: '+' –within the same HA or LA line, between groups subjected to acute or chronic administration of PTZ (modification of seizures intensity by repeated injections with PTZ). '$' considering effect of repeated PTZ dosing, between individuals of HA and LA mice (interaction between line type and PTZ treatment regimen). '×' considering effect of NLX or morphine, between individuals of HA and LA mice (interaction between line type and NLX or morphine). '@' within the same line type, considering the effect of NLX or morphine, between acute and chronic administration of PTZ (interaction between PTZ treatment regimen and NLX or morphine). 'Φ' between HA and LA line, considering changes in NLX or morphine effect, between acute and chronic administration of PTZ (interactions among mouse line type, PTZ treatment regimen and NLX or morphine). Subscripts 1, 2 and 3 denotes significance of comparisons fixed at 0.001; 0.01 and 0.05, respectively.

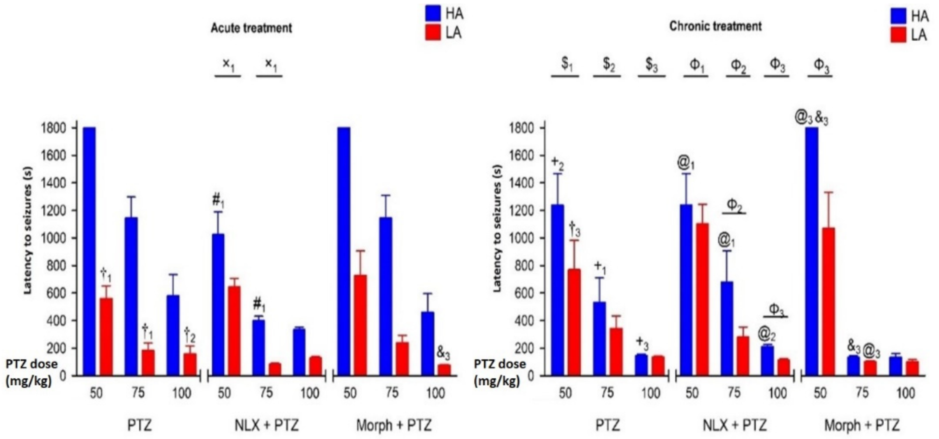

Figure S4. Changes in naloxone-mediated inhibition or morphine-induced stimulation of opioid system on latency to seizures onset in HA and LA mice receiving ip. either a single boost of PTZ (acute treatment) or repeated doses of PTZ. NLX or morphine at a dose of 27.50 μmol/kg were administered acutely or chronically 30 min before PTZ injection. Seizures were kindled every day by ip. injection of PTZ at a dose of 25 mg/kg. Each experimental group consisted of 8–10 mice. Subsequent symbols denote significance of comparisons: within LA or HA line, between subgroups receiving PTZ vs. mice groups additionally injected with NLX (‘#’) or morphine (‘&’) to modify PTZ effect. ‘†’ - between subgroups of HA and LA mice, within each PTZ dose (epileptogenic effect of PTZ). ‘+’ –within the same HA or LA line, between groups subjected to acute or chronic administration of PTZ (modification of seizures intensity by repeated injections with PTZ). ‘$’ considering effect of repeated PTZ dosing, between individuals of HA and LA mice (interaction between line type and PTZ treatment regimen). ‘×’ considering effect of NLX or morphine, between individuals of HA and LA mice (interaction between line type and NLX or morphine). ‘@’ within the same line type, considering the effect of NLX or morphine, between acute and chronic administration of PTZ (interaction between PTZ treatment regimen and NLX or morphine). ‘Φ’ between HA and LA line, considering changes in NLX or morphine effect, between acute and chronic administration of PTZ (interactions among mouse line type, PTZ treatment regimen and NLX or morphine). Subscripts $_1$, $_2$ and $_3$ denotes significance of comparisons fixed at 0.001; 0.01 and 0.05, respectively.
